# Chlorogenic Acid Combined with *Lactobacillus plantarum* 2142 Reduced LPS-Induced Intestinal Inflammation and Oxidative Stress in IPEC-J2 Cells

**DOI:** 10.1371/journal.pone.0166642

**Published:** 2016-11-18

**Authors:** Orsolya Palócz, Erzsébet Pászti-Gere, Péter Gálfi, Orsolya Farkas

**Affiliations:** Department of Pharmacology and Toxicology, University of Veterinary Medicine, Budapest, Hungary; University of British Columbia, CANADA

## Abstract

This study was carried out to investigate protective effect of chlorogenic acid against lipopolysaccharide-induced inflammation and oxidative stress in intestinal epithelial cells. As a marker of inflammatory response, IL-6, IL-8, TNF-α mRNA and protein levels, furthermore, COX-2 mRNA level were followed up. Intracellular redox status and extracellular H_2_O_2_ level were also monitored by two fluorescent assays (DCFH-DA, Amplex Red). Moreover, the effect of gut microbiota metabolites in the above mentioned processes was taken into account in our model using *Lactobacillus plantarum* 2142 bacterial strain. Our data revealed that chlorogenic acid had significant lowering effect on the inflammatory response. Treatment with chlorogenic acid (25–50 μM) significantly decreased gene expression and concentration of proinflammatory cytokines IL-6 and IL-8 compared to LPS-treated cells. COX-2 and TNF-α mRNA levels were also reduced. Furthermore, chlorogenic acid reduced the level of reactive oxygen species in IPEC-J2 cells. Simultaneous application of chlorogenic acid and *Lactobacillus plantarum* 2142 supernatant resulted protective effect against LPS-induced inflammation and oxidative stress as well.

## Introduction

The gut has two equally important functions, i.e. the digestion and host defence. Impact of environmental stress and infections caused by pathogens could lead to dysfunctional epithelium; furthermore, chronic inflammatory disorders (e.g. Crohn’s disease and ulcerative colitis) and other kind of diseases could be developed such as type 1 diabetes [1,2,3]. Many studies have shown that the chronic phase of inflammation is clearly associated with the up-regulation of certain enzymes (e.g. COX-2), overproduction of reactive oxygen species (ROS), proinflammatory cytokines and other signalling proteins in the affected tissues. The fact that presence of chronic inflammation in the gastrointestinal system is correlated with increased cancer risk is widely known, because of sharing the same signal transduction pathways [4].

Well-functioning gut has a key importance in prevention of gastrointestinal diseases. Gut health could be improved and maintained with proper selection of food and food additives which could help to prevent pathogen invasion, promote ROS homeostasis, furthermore to stimulate growth of useful microorganisms [5].

**Fig 1:**
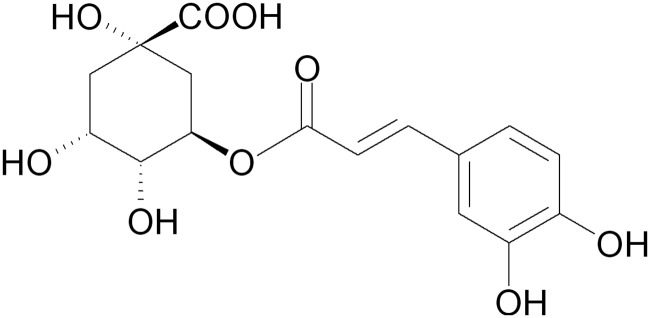
Structure of chlorogenic acid.

Plant-derived polyphenols are part of the balanced human diet [6], and they could be also found in many food products. Polyphenols are mainly classified into phenolic acids and flavonoids. A major class of the phenolic acids is hydroxycinnamic acids, and chlorogenic acid (Fig 1) is the major representative of the above mentioned group [7]. It is known as one of the most abundant polyphenols in the nature as well as in the human diet. Chlorogenic acid is a major polyphenol in coffee; coffee drinkers consume up to 1 g chlorogenic acid/day. It is widespread in fruits and vegetables as well [8], including apples, pears, carrots, tomatoes and sweet potatoes. Chlorogenic acid is hydrolized by gut microflora into various aromatic acid metabolites including caffeic acid and quinic acid [7,9].

Many studies have pointed out the positive effects of chlorogenic acid under the condition of inflammation. However, most of these studies were investigated on immune or on other non-intestinal cells. For instance, in human lung carcinoma cells, chlorogenic acid evoked up-regulation of cellular antioxidant enzymes and suppressed ROS-mediated nuclear factor kappa B (NF-κB), AP-1 and mitogen-activated protein kinase activations as well [10]. *In vivo* studies also underline the significance of chlorogenic acid in reduction of the inflammation. Intraperitoneal administration of chlorogenic acid decreased lipopolysaccharide (LPS) -induced mammary gland injury and inflammatory cell infiltration in the mammary gland, down-regulate the production of TNF-α, IL-6 and IL-1β in a dose dependent manner [11]. In addition, chlorogenic acid showed anti-hepatotoxic effect on LPS-treated mice by suppression of the mRNA levels of TLR4, TNF-α and the NF-κB p65 subunit [12].

The absorption of polyphenols and other plant-derived compounds could be influenced by many parameters mainly depending on the gut conditions and the food matrix [13,14]. The intestinal microflora could mediate the metabolism and bioactivation of polyphenols, so it could enhance their possible positive effects. For instance, chlorogenic acid is not reported to have significant bioavailability until it reaches the colon, where the microbiota rapidly hydrolyses it to liberate caffeic acid [9]. Metabolites of probiotic bacteria also have a proven anti-inflammatory effect [15,16]. Furthermore, unabsorbed polyphenols in the colon could influence the constitution and quality of microbiota. Therefore, *in vitro* intestinal models should be completed probiotic bacteria in order to understand better the anti-inflammatory processes in the gut.

The main aim of this study is to investigate the possible protective effects of chlorogenic acid in intestinal epithelial cells under the condition of inflammation. To our knowledge, this is the first report, which describes the effect of chlorogenic acid in intestinal inflammation and oxidative stress using healthy intestinal epithelial cell line. Moreover, simultaneous application of chlorogenic acid and metabolites of a probiotic bacterial strain was also performed at first time.

## Materials and Methods

### Chemicals

Chlorogenic acid (95% ≥) and LPS (derived from *Salmonella enterica* ser. Typhimurium, suitable for cell culture) were purchased from Sigma–Aldrich (Steinheim, Germany).

### Cell line and culture conditions

The non-transformed porcine intestinal epithelial cell line IPEC-J2, originally isolated from jejunal epithelia of a neonatal unsuckled piglet [17], was a kind gift of Dr. Jody Gookin, Department of Clinical Sciences, College of Veterinary Medicine, North Carolina State University, Raleigh, NC, USA. IPEC-J2 cells were grown and maintained in complete medium, which consisted of a 1:1 mixture of Dulbecco's Modified Eagle's Medium and Ham's F-12 Nutrient Mixture (DMEM/F12) (plain medium) supplemented with 5% foetal bovine serum (FBS), 5 μg/ml insulin, 5 μg/ml transferrin, 5 ng/ml selenium, 5 ng/ml epidermal growth factor and 1% penicillin-streptomycin (all from Fisher Scientific, Loughborough, UK). Cells were grown at 37°C in a humidified atmosphere of 5% CO_2_. Cell cultures were tested by PCR and they were found to be free of *Mycoplasma* contamination.

For the experiments, IPEC-J2 cells between passages 42–48 were seeded onto six-well Transwell Polyester membrane inserts (Corning Inc., Corning, NY, USA), the latter coated with 8 μg/cm^2^ rat tail collagen type I (Sigma-Aldrich, Steinheim, Germany), at a density of 1.5x10^5^ cells/ml (the volume of complete medium was 1.5 ml on the apical side and 2.5 ml on the basolateral side per well according to the manufacturer’s instructions). Cells were allowed to adhere for 24 h before being washed and re-fed every other day until confluence was reached. Transepithelial electrical resistance (TEER) measurement of monolayers was performed on alternate days after seeding, from day 5 to 21 of culture, using an EVOM Epithelial Tissue Volt/Ohmmeter (World Precision Instruments, Berlin, Germany).

### *Lactobacillus plantarum* 2142 strain and spent culture supernatant

*Lactobacillus plantarum* 2142 (Lp 2142, Culture Collection of the Institute of Dairy Microbiology, Agricultural Faculty of Perugia University, Perugia, Italy) was grown in DeMan, Rogosa, Sharpe (MRS) broth. Inoculation was accomplished with a stationary culture of probiotical strain (1% inoculum) and the bacteria were grown for 24 h at 37°C. They were subcultured at least twice before experiments. Spent culture supernatant (SCS) were prepared by centrifugation of the bacterial suspension (with final bacterial concentrations of 10^9^ CFU/ ml) at 3000 g at 5°C for 10 min. Centrifuged culture supernatant was then passed through a sterile 0.22 μm pore size filter unit.

### Neutral Red uptake assay for cell viability

Influence of chlorogenic acid on the viability of IPEC-J2 cells in different concentrations (25, 50 and 100 μM) was tested. Chlorogenic acid was dissolved and diluted in cell culture medium. IPEC-J2 cells were seeded in a 96-well plate and incubated with chlorogenic acid for 1, 4 and 24 h, respectively. Viability of IPEC-J2 cells was measured after 24 h of treatment by Neutral Red uptake assay as described by Repetto et al. [18].

### Treatment of cells with LPS and test compounds

Before treatment, confluent monolayers of the IPEC-J2 cells were washed with plain medium. LPS solutions were prepared freshly prior to each experiment. LPS was added in plain medium at 10 μg/ml on the apical side of the IPEC-J2 layer [19]. After 1 h incubation with LPS and test compounds, cells were washed with plain medium and cultured for additional 1 h for PCR studies. TEER measurements were performed both before and after the LPS treatment.

### Determination of intracellular redox status and extracellular H_2_O_2_ levels in IPEC-J2 cells

Extracellular ROS measurement was based on the detection of H_2_O_2_ using the Amplex Red Hydrogen Peroxide Assay Kit (Invitrogen, Molecular Probes) keeping the IPEC-J2 cells on the 96-well plate. In the presence of horseradish peroxidase (HRP), Amplex Red reacts with H_2_O_2_ in a 1:1 stoichiometry producing a highly fluorescent resorufin [20].

IPEC-J2 cells were treated with LPS (10 μg/ml) in phenol-red free DMEM and the H_2_O_2_ concentrations in the medium were determined using the working solution of 100 μM Amplex Red reagent and 0.2 U/ml HRP. H_2_O_2_ determination was also performed after 1 h LPS treatment and 24 h incubation with phenol-red free DMEM. After 60 min incubation with the dye at room temperature the quantitative analyses of H_2_O_2_ contents were accomplished, the excitation wavelength was set at 560 nm and emission was measured at 590 nm (Victor X2 2030 fluorometer, Perkin Elmer, Waltham, MA, USA).

Measurement of perturbances in intracellular redox state of IPEC-J2 cells was carried out using 2’,7’-dichloro-dihydro-fluorescein diacetate (DCFH-DA) dye (Sigma-Aldrich, Budapest, Hungary). DCFH-DA is oxidized to the highly fluorescent form dichloro- fluorescein (DCF) by the intracellular ROS [21]. IPEC-J2 cells were treated with LPS (10 μg/ml) in phenol-red free DMEM for 1 h in phenol-red free DMEM. A working solution of 10 μM DCFH-DA was added to IPEC-J2 cells and incubation with the dye continued for 30 minutes. The cells were then washed, scraped and centrifuged for 10 minutes at 4500 rpm at 4°C. The fluorescence of the supernatant was measured with the Victor X2 2030 fluorometer. Excitation wavelength was set at 480 nm and emission wavelength read at 530 nm.

### Quantitative real time PCR

One hour after the 1 h LPS treatment, culture medium was removed and 1 ml of ice-cold TRIzol reagent (Invitrogen, Carlsbad, CA, USA) was added to the IPEC-J2 samples. Samples were collected and kept at -80°C until further processing. Total RNA was isolated from the cells according to the manufacturer’s instructions. To prevent DNA contamination, the isolated RNA (2 μg) was treated with AMP-D1 DNase I (Sigma). Quantity, A_260_/A_280_ and A_260_/A_230_ ratios of the extracted RNA were determined using a NanoDrop ND-1000 Spectrophotometer (Thermo Scientific, Wilmington, USA). Quality and quantity control of the isolated RNA was carried out both before and after the DNase treatment.

Synthesis of the first strand of cDNA from 1000 ng of total RNA was achieved using RevertAid H Minus First Strand cDNA Synthesis Kit (Fermentas, St. Leon-Roth, Germany) according to the manufacturer’s recommendations, using the random hexamer as a priming method. Quantitative real-time PCR (qRT-PCR) was performed using the iQ SYBR Green Supermix kit (BioRad, Hercules, CA, USA) on the MiniOpticon System (BioRad). The cDNA was diluted 5-fold, before equal amounts were added to duplicate qRT-PCR reactions. Tested genes of interest were IL-8 [22], IL-6 [23], TNF-α [24] and COX-2 [19]. Hypoxanthine phosphoribosyltransferase (HPRT) [25] and Cyclophilin-A (CycA) [24] were used as reference genes. Primer sequences are listed in Table 1.

**Table 1:** Sequence of primer sets used for quantitative real-time.

Gene symbol	Accession number	Primer sequences	Product size (bp)	Efficiency	R^2^
IL-8	NM_213867	F 5’AGAGGTCTGCCTGGACCCCA-3’	126	1.972	0.999
		R 5’-GGGAGCCACGGAGAATGGGT-3’			
IL-6	NM_214399	F 5’-TTCACCTCTCCGGACAAAAC-3’	122	1.970	0.995
		R 5’-TCTGCCAGTACCTCCTTGCT-3’			
TNF-α	NM_214022	F 5’-TTCCAGCTGGCCCCTTGAGC-3’	146	1.873	0.982
		R5’-GAGGGCATTGGCATACCCAC-3’			
Cox-2	NM_214321	F 5’-AGAAGCGAGGACCAGCTTTC-3’	215	1.905	0.981
		R 5’-AAAGCGGAGGTGTTCAGGAG-3’			
CycA	NM_214353	F 5’-GCGTCTCCTTCGAGCTGTT-3’	160	1.907	0.998
		R 5’-CCATTATGGCGTGTGAAGTC-3’			
HPRT	NM_001032376	F 5’-GGACTTGAATCATGTTTGTG-3’	91	1.963	0.997
		R 5’-CAGATGTTTCCAAACTCAAC-3’			

For each PCR reaction, 2.5 μl cDNA was added directly to a PCR reaction mixture, set to a final volume of 25 μl, containing 1x concentrated iQ SYBR Green Supermix and 0.2 μM of the appropriate primers. The thermal profile for all reactions was 3 min at 95°C, then 40 cycles of 20 sec at 95°C, 30 sec at 60°C, and 30 sec at 72°C. At the end of each cycle, the fluorescence monitoring was set for 10 seconds. Each reaction was completed with a melting curve analysis to ensure the specificity of the reaction. In order to determine the efficiencies of the PCR reactions, standard curves were obtained for each target and reference gene, using serial dilutions of a reference cDNA. Real-time PCR efficiencies (E) were calculated according to the equation: E = 10(-1/slope). To determine the stability of the reference genes, the geNorm (version 3.5) was used.

### IL-8, IL-6 and TNF-α ELISA

After 1h treatment, IPEC-J2 cells were incubated with cell culture medium. Culture media were collected after 6, 12 and 24 h, respectively. The samples were centrifuged (245 x g, 10 min) and cytokine concentrations were measured. Level of IL-8, IL-6 and TNF-α secretion (pg/ml) was determined by porcine-specific ELISA Kits (Thermo Scientific, MA, USA) according to the manufacturer’s instructions.

### Statistics

Relative gene expression levels of the genes of interest were calculated by the Relative Expression Software Tool (REST) 2009 Software (Qiagen GmbH, Hilden, Germany). Statistical analysis of other data was performed with STATISTICA 12 software (StatSoft Inc., Tulsa, USA). Differences between means were evaluated by two-way ANOVA, with data of normal distribution, and homogeneity of variances was confirmed. To compare treated groups to controls Dunnett post-hoc test was used. For the comparison of different treatments we used Fisher LSD test. Level of significance was set at P<0.05. All values were expressed as means ± standard deviations.

## Results

### Viability of IPEC-J2 cells after chlorogenic acid treatment

**Fig 2:**
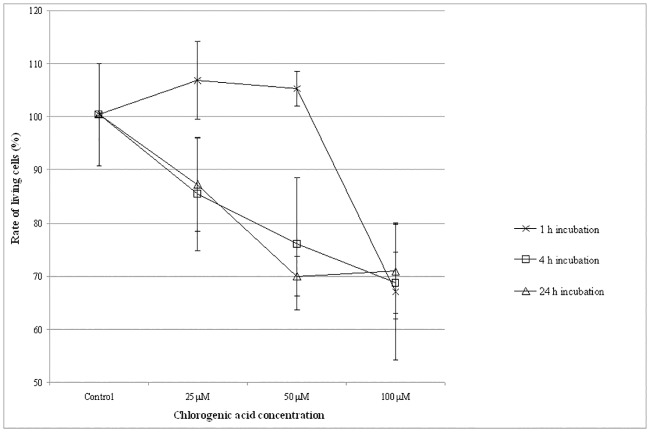
Rate of living IPEC-J2 cells after chlorogenic acid treatment. Cell viability was measured by Neutral Red uptake method. Effect of chlorogenic acid on cell viability was studied in a dose-(CGA concentrations: 25 μM, 50 μM and 100 μM) and in a time-dependent manner (treatment time: 1 h, 4 h and 24 h). Data are shown as means + standard deviations. CGA: chlorogenic acid.

Viability of IPEC-J2 cells was monitored after chlorogenic acid treatment (Fig 2). Neutral Red uptake assay showed that at 25 μM treatment dose for 1 h did not reduce the number of viable enterocytes significantly. The same could be observed when IPEC-J2 cells were incubated with 50 μM chlorogenic acid for 1 h. Thirty-three % of the IPEC-J2 cells were killed by 1 h treatment when chlorogenic acid was applied at 100 μM concentrations. When cells were treated for 4 h with chlorogenic acid using different concentrations, reduced viability was measured compared to the control samples. In case of 25 μM chlorogenic acid treatments, the number of viable cells decreased to 85.4±10.6%, while living cell rate decreased to 76.0±12.5% when 50 μM chlorogenic acid solutions were used. Viability was reduced to 68.8±5.7% at 100 μM chlorogenic acid exposure. After 24 h treatment of chlorogenic acid (25, 50 and 100 μM), significantly reduced viability of IPEC-J2 cells was also detected (rate of living cells decreased to 87.3±8.9%, 70.0±3.7% and 71.1±9% respectively, compared to the control samples). On the basis of the above mentioned data, it seemed to be safe to use chlorogenic acid in 25–50 μM concentration range in further experiments. Treatment period was chosen to 1 h. Viability data are available in S1 Table.

### Inflammatory response in IPEC-J2 cells after LPS treatment

Relative gene expressions of TNF-α, IL-6, IL-8 and COX-2 in LPS-triggered IPEC-J2 cells were measured. LPS treatment significantly increased IL-6 (P = 0.044), IL-8 (P = 0.001) and TNF-α (P = 0.018), relative gene expression levels in the enterocytes. Significant up-regulation of COX-2 gene was also detected after 1 h 10 μg/ml LPS administration (P = 0.012). Quantity of IL-8, IL-6 and TNF-α production was also followed up. Significant increase of IL-8 concentration was observed in the IPEC-J2 cells 24 h after of LPS treatment (P = 0.0010). There was no significant difference in the IL-8 levels of LPS treated samples and controls in case of 6 h and 12 h samples. The 10 μg/ml LPS treatment caused elevated IL-6 level 6 h after LPS treatment (P<0.0001). When sampling was performed 12 and 24 h after LPS treatment, respectively, the IL-6 concentrations in the LPS treated samples did not differ significantly from the controls. However, TNF-α secretion was observed neither in the LPS-treated nor in the control IPEC-J2 cells.

In order to verify the integrity of the IPEC-J2 cell layer, TEER values between apical and basolateral compartment were measured. Experiments were performed with confluent polarized IPEC-J2 cells with high TEER values. The integrity of the cell monolayer was not altered after LPS treatment.

### Effect of chlorogenic acid on the inflammatory markers after LPS-treatment

**Fig 3:**
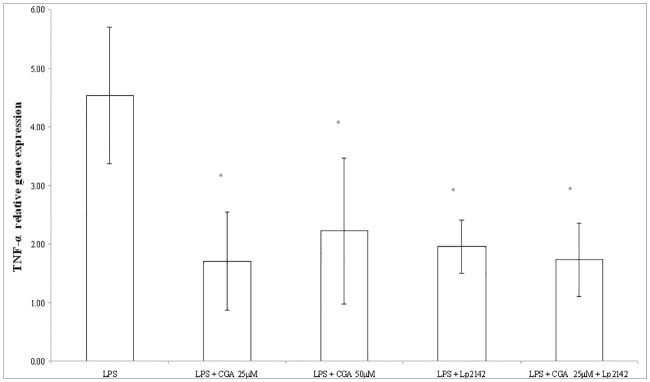
Relative gene expression of TNF-α in IPEC-J2 cells exposed to LPS treatment (at 10 μg/ml; 1 h treatment). Effect of CGA (25 μM, 50 μM), Lp 2142 SCS (13.3%) and combined treatment (CGA 25 μM and Lp 2142 SCS on the TNF-α mRNA levels (n = 3-4/group; *P<0.05). Data are shown as means + standard deviations. CGA: chlorogenic acid, Lp 2142: *Lactobacillus plantarum* 2142.

**Fig 4:**
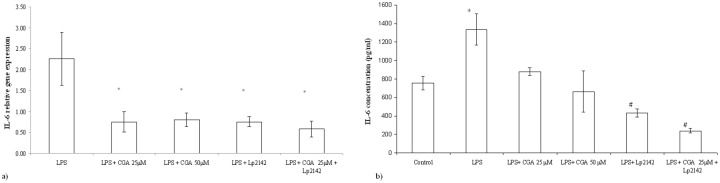
(a) Relative gene expression and (b) concentration of IL-6 in IPEC-J2 cells exposed to LPS treatment (at 10 μg/ml; 1 h treatment). Effect of CGA (25 μM, 50 μM), Lp 2142 SCS (13.3%) and combined treatment (CGA 25 μM and Lp 2142 SCS on the IL-6 mRNA levels (n = 3-4/group; *P< 0.05). Data are shown as means + standard deviations. CGA: chlorogenic acid, Lp 2142: *Lactobacillus plantarum* 2142.

**Fig 5:**
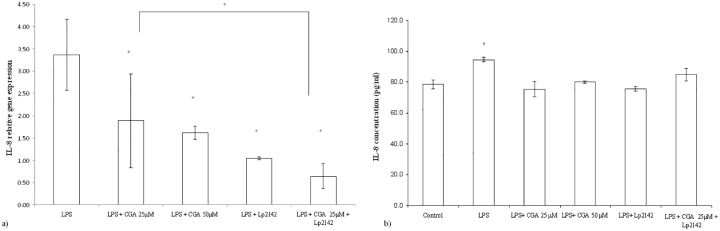
(a) Relative gene expression and (b) concentration of IL-8 in IPEC-J2 cells exposed to LPS treatment (at 10 μg/ml; 1 h treatment). Effect of CGA (25 μM, 50 μM), Lp 2142 SCS (13.3%) and combined treatment (CGA 25 μM and Lp 2142 SCS on the IL-8 mRNA levels (n = 3-4/group; *P< 0.05). Data are shown as means + standard deviations. CA: chlorogenic acid, Lp 2142: *Lactobacillus plantarum* 2142.

**Fig 6:**
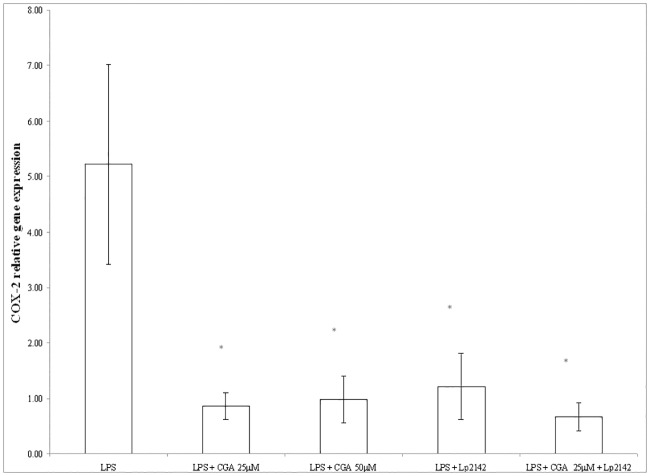
Relative gene expression of COX-2 in IPEC-J2 cells exposed to LPS treatment (at 10 μg/ml; 1 h treatment). Effect of CGA (25 μM, 50 μM), Lp 2142SCS (13.3%) and combined treatment (CGA 25 μM and Lp 2142SCS on the COX-2 mRNA levels (n = 3-4/group; *P< 0.05). Data are shown as means + standard deviations. CGA: chlorogenic acid, Lp 2142: *Lactobacillus plantarum* 2142.

At 50 μM chlorogenic acid treatment dose, relative gene expression of IL-6 significantly decreased compared to LPS-treated cells (P = 0.0009). Chlorogenic acid in lower treatment dose (25 μM) also resulted a reduced IL-6 mRNA level (P = 0.00072). Chlorogenic acid resulted significant reduction in the IL-8 gene expression in case of both 25 and 50 μM (P = 0.034; P = 0.013) treatments. TNF-α mRNA level was leveraged by 25 μM and 50 μM chlorogenic acid treatment (P = 0.008; P = 0.027 respectively) as well. Figs 3, 4a and 5a show the effect of chlorogenic acid on proinflammatory cytokine gene expressions. Chlorogenic acid treatment (25 and 50 μM) caused significant reduction in the mRNA level of COX-2 (P = 0.008; P = 0.027 respectively). Influence of chlorogenic acid on COX-2 relative gene expression could be seen in Fig 6.

The increased interleukin concentrations were reduced after chlorogenic acid treatment. Both 25 μM (P = 0.0002) and 50 μM (P = 0.0023) chlorogenic acid exposure decreased significantly the IL-8 cytokine concentrations. The same phenomenon could be observed in case of IL-6 protein levels (P = 0.0002 in case of 25 μM, P<0.0001 in case of 50 μM chlorogenic acid treatment). There was no significant difference between the effects of the two concentrations. Figs 4b and 5b show the effect of chlorogenic acid on IL-8 and IL-6 protein levels. Relative gene expression data and cytokine concentration values are available in S2 Table.

### Effect of simultaneous chlorogenic acid and Lp 2142 spent culture supernatant treatment on IPEC-J2 cells

Because there was no significant difference between the anti-inflammatory effect of 25 μM and 50 μM chlorogenic acid treatment in reducing inflammatory gene expression, simultaneous application of the chlorogenic acid and Lp 2142 was carried out only using 25 μM polyphenol concentration. Metabolites of Lp 2142 caused significant reduction in the level of IL-6 (P = 0.00073), IL-8 (P = 0.0018), TNF-α (P = 0.0096) and COX-2 (0.0096) as well. Simultaneous treatment led also to the significant reduction of IL-6 (P = 0.0003), IL-8 (P = 0.0018), TNF- α (P = 0.0085) and COX-2 (P = 0.0085) mRNA levels as well. Effectiveness in treatment LPS-evoked inflammation of chlorogenic acid, Lp 2142 and combined treatment in IPEC-J2 cells was compared. The two factors in the experiments were the presence and the lack of the Lp 2142 supernatant. There was significant difference (P = 0.033) between the effect of chlorogenic acid and combined treatment only in case of IL-8 mRNA values. In the presence of Lp 2142 supernatant the treatment was more effective than without it.

Both IL-6 (P<0.0001) and IL-8 (P = 0.0003) concentrations were significantly reduced by simultaneous treatment of chlorogenic acid and Lp 2142. The combined treatment was more effective in reducing IL-6 concentration than in case of 25 μM (P<0.0001) and 50 μM (P = 0.0003) chlorogenic acid treatment, respectively.

### ROS production in IPEC-J2 cells after chlorogenic acid treatment

**Fig 7:**
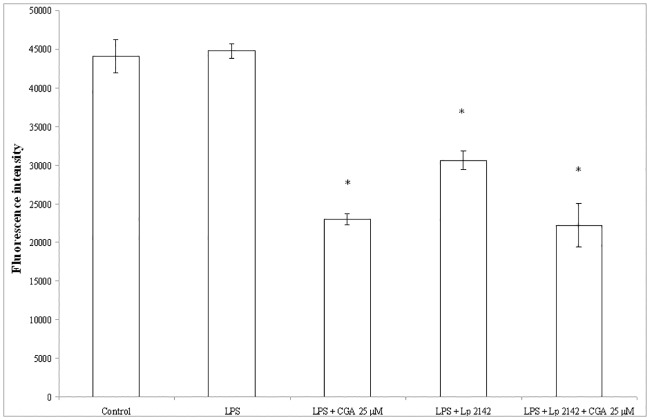
Level of extracellular H_2_O_2_ in IPEC-J2 cells exposed to CGA treatment (25 and 50 μM; 24 h). Effect of CGA on the relative extracellular H_2_O_2_ levels (n = 3-4/group; *P< 0.05). Fluorescence measurement was performed by Amplex Red method. Fluorescence was detected 24 h after CGA treatment. Data are shown as means + standard deviations. CGA: chlorogenic acid, Lp 2142: *Lactobacillus plantarum* 2142.

**Fig 8:**
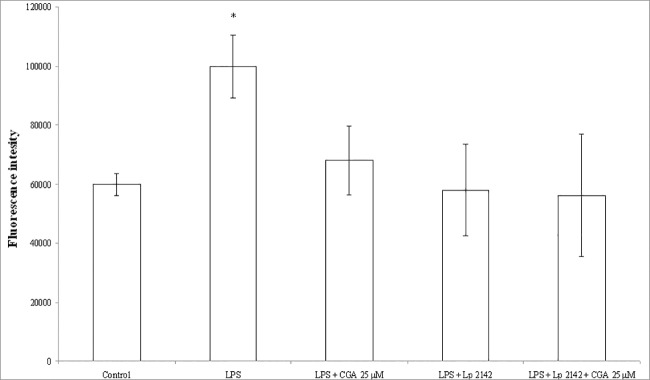
Intracellular redox status in IPEC-J2 cells exposed to CGA treatment (25 and 50 μM; 24 h). Effect of CGA on the relative intracellular ROS levels (n = 3-4/group; *P< 0.05). Fluorescence measurement was performed by DCFH-DA method. Fluorescence was detected 24 h after CGA treatment. Data are shown as means + standard deviations. CGA: chlorogenic acid, Lp 2142: *Lactobacillus plantarum* 2142.

Fig 7 represents the relative extracellular H_2_O_2_ level in the enterocytes after chlorogenic acid and Lp 2142 treatment. The response of the detection system was not significantly affected by 10 μg/ml LPS (Fig 7). Contrarily, relative intracellular level of ROS was increased significantly 24 h after 10 μg/ml LPS exposure (P = 0.024), when DCFH-DA method was applied (Fig 8). Chlorogenic acid showed reducing effect on the rate of extracellular H_2_O_2_ and intracellular ROS production as well. Both extracellular H_2_O_2_ and intracellular ROS levels were significantly decreased after Lp 2142 supernatant (13.3%) treatment. The ROS-decreasing effect did not differ significantly, when 25 μM chlorogenic acid and Lp 2142 were applied simultaneously. Data are available in S3 Table.

Effect of chlorogenic acid in the lack of LPS treatment was also followed up. It was found, that extracellular H_2_O_2_ level was reduced by both 25 μM and 50 μM chlorogenic acid treatment. Intracellular ROS level was affected only by 25 μM chlorogenic acid treatment. Lp 2142 exposure caused a slight reduction in extracellular H_2_O_2_, while did not reduce the intracellular ROS level (data not shown).

## Discussion

The intestinal epithelium participates in the mucosal immune response against pathogen bacteria and in the regulation of mucosal tissue homeostasis [26]. Under the condition of inflammation, a variety of transcription factors (e.g. NF-κB, AP-1, Nrf2) and gene became activated triggering the production of inflammatory cytokines, chemokines and resulted the presence of lipid mediators generated by COX-2. Furthermore, the activation of the above mentioned transcription factors induce the change of the cellular redox status shifting the equilibrium to the ROS predominance [27]. On the other hand, bacterial invasion also could change the redox state which could lead to initiation of various signalling pathways and regulate the transcriptional and post-transcriptional events that control inflammatory gene expression.

In order to avoid the above mentioned imbalance of redox homeostasis gut health could be improved and maintained with proper selection of feed additives. Nowadays there is a growing interest to replace the use of antibiotics by different natural alternatives and chlorogenic acid seems as a promising compound. There are some papers discussing the anti-inflammatory effect of chlorogenic acid in the gastrointestinal system. However, it is recommendable to compare the results of these publications carefully. In many reports cancerous intestinal cell lines such as Caco-2 were used [7,28], where the cell model system does not describe *in vivo* conditions of the gut properly. Ethical considerations of animal well-being as well as cost reduction require limited use of experimental animals as much as possible. Beside of the current transformed intestinal cell lines it is worthy to apply healthy cell lines to mimic and study the *in vivo* physiological and pathophysiological mechanisms in the small intestine such as using IPEC-J2. It is a non-carcinogenic swine intestinal epithelial cell line, which was successfully used in the last decade to model the above mentioned processes [17,29]. Further advantage of pig intestinal cell model is its similarity with human gut, which could lead to the use of IPEC-J2 also in human medical research and food science.

It is worthy to note, that research data of antioxidant and anti-inflammatory activities of chlorogenic acid came often from studies in which plant extracts are used as test objects. In these studies, chlorogenic acid is often identified as main effective compound of a complex infusion. For instance, the anti-inflammatory effect of an extract from *Cymbopogon citratus* (CC) in human macrophages was demonstrated. The results showed that the leaf extract of CC inhibits LPS-induced cytokine expression through NF-κB pathway [30]. In addition, *Artemisia annua* extract was applied on Caco-2 cells in order to reduce intestinal inflammation. The UPLC-DAD-MS/MS measurements identified chlorogenic acid and rosmarinic acid as the main phenolic compounds in the extract, which significantly reduced the secretion of pro-inflammatory cytokines IL-8 and IL-6 in the intestinal cells [31]. Effect of chlorogenic acid and its metabolites in enterocytes were also studied by Shin et al. [28]. Caco-2 cells were triggered by combined stimulation with TNF-α and H_2_O_2_, and chlorogenic acid significantly decreased the IL-8 production. These data are in accordance with our results proving the role of chlorogenic acid in reduction of proinflammatory cytokine levels.

To the best of our knowledge, there are no available scientific data about the effect of chlorogenic acid on the COX-2 expression in intestinal cells. Our data show, that COX-2 mRNA level is effectively reduced by chlorogenic acid treatment. Anti-inflammatory effect of chlorogenic acid was tested in LPS-triggered RAW 264.7 macrophage cells [32]. Authors observed the attenuation of various inflammation markers such as COX-2, TNF-α, IL-1β and IL-6. In the study of Shan et al [33], decrease in COX-2 mRNA level in LPS-stimulated macrophages was established after chlorogenic acid treatment via attenuating the activation of NF-κB and JNK/AP-1 pathways. On the other hand, chlorogenic acid treatment did not influence the phosphorylation of ERK1/2 and p38.

In our study, oxidative stress markers were also followed. It was found, that level of extracellular H_2_O_2_ detected by Amplex Red method was not affected by 10 μg/ml LPS treatment. LPS in higher concentration (50 μg/ml) increased intracellular H_2_O_2_ level significantly, however, IPEC-J2 cells are irreversible damaged [19]. Still, it is important to examine the impact of chlorogenic acid on IPEC-J2 cells, because of its possible pro-oxidant effect. High-dose chlorogenic acid (7 mg/kg BW) increased the generation of peroxides in the venular walls in rats, moreover, an increase in maleic dialdehyde, myeloperoxidase, inflammatory cytokines and NADPH oxidase activities could be also observed [34]. In the same study, low-dose chlorogenic acid treatment did not affect negatively the redox state of animals. Our cell culture data showed that chlorogenic acid in lower dose (25–50 μM) led to the reduction of H_2_O_2_ level in enterocytes. Chlorogenic acid in higher dose (100 μM) resulted significant cell death, therefore it was not applied to treat IPEC-J2 cells.

Nevertheless, it is worthy to apply further ROS-measuring techniques in order to have a more complex characterization of redox status in enterocytes. While Amplex Red method is used to detect H_2_O_2_ in biological samples, DCFH-DA probe measures hydroxyl, peroxyl and other ROS within the cells [35]. Moreover, LPS treatment caused elevated ROS level in the IPEC-J2 cells followed by DCFH-DA method, which opens an opportunity to study the protective effect of polyphenols in oxidative stress caused by pathogenic bacteria. Antioxidant activity and ability to reduce intracellular ROS of chlorogenic acid was also tested on human intestinal HT-29 cell line and using DCFH-DA probe [36]. Compared with the methanol-stimulated control a significant decrease in ROS levels was observed.

In our previous works, secreted bioactive metabolites of lactobacilli were proved to be effective in the treatment of intestinal inflammation on *in vitro* intestinal models [15,16]. We demonstrated that spent culture supernatant (13.3%) of Lp 2142 was effective in decreasing IL-8 and TNF-α mRNA level in IPEC-J2 cultures after LPS treatment. Now, suppression of IL-6 and COX-2 mRNA levels after LPS exposure was observed as well. Furthermore, Lp 2142 significantly attenuated the LPS-induced oxidative stress in IPEC-J2 cells. The human diet contains several bioactive compounds, therefore, the biological activity often cannot be assigned to a single compound, but potential synergistic effects should be taken into account. In our study, mRNA levels of inflammatory markers such as cytokines and COX-2 were reduced significantly compared to the LPS-treated samples and did not differ significantly from the control values. The same phenomenon could be observed in case of redox status in enterocytes followed by DCFH-DA fluorescent probe. Simultaneous treatment with chlorogenic acid and Lp 2142 metabolites resulted also a significant decrease in proinflammatory marker mRNA and ROS levels. Synergistic effect between Lp 2142 metabolites and chlorogenic acid treatment could be not observed, however, the effectiveness of tested compounds in itself caused the reduction of inflammatory markers to the control level in most cases.

In conclusion, the present study demonstrated that chlorogenic acid could be a promising compound in the prevention and treatment of intestinal inflammation in human as well in veterinary medicine. Further research should focus on developing *in vitro* model systems working with living microorganisms, in order to study the effect of metabolites of chlorogenic acid and other natural polyphenols.

## Supporting Information

S1 Table Viability of IPEC-J2 cells after chlorogenic acid treatment. (DOCX)

S2 Table Relative gene expression and protein concentrations of inflammatory markers. (DOCX)

S3 Table Fluorescence intensity values in the ROS measurements. (DOCX)

## References

[1] Macdonald TT, Monteleone G (2005) Immunity, inflammation, and allergy in the gut. Science 307: 1920–1925. 10.1126/science.1106442 15790845

[2] Sonier B, Patrick C, Ajjikuttira P, Scott FW (2009) Intestinal Immune Regulation as a Potential Diet-Modifiable Feature of Gut Inflammation and Autoimmunity. International Reviews of Immunology 28: 414–445. 10.3109/08830180903208329 19954357

[3] Wang DZ, DuBois RN, Richmond A (2009) The role of chemokines in intestinal inflammation and cancer. Current Opinion in Pharmacology 9: 688–696. 10.1016/j.coph.2009.08.003 19734090 PMC2787713

[4] Ullman TA, Itzkowitz SH (2011) Intestinal Inflammation and Cancer. Gastroenterology 140: 1807–U1148. 10.1053/j.gastro.2011.01.057 21530747

[5] Rahman I, Biswas SK, Kirkham PA (2006) Regulation of inflammation and redox signaling by dietary polyphenols. Biochemical Pharmacology 72: 1439–1452. 10.1016/j.bcp.2006.07.004 16920072

[6] Sergent T, Piront N, Meurice J, Toussaint O, Schneider YJ (2010) Anti-inflammatory effects of dietary phenolic compounds in an in vitro model of inflamed human intestinal epithelium. Chemico-Biological Interactions 188: 659–667. 10.1016/j.cbi.2010.08.007 20816778

[7] Sato Y, Itagaki S, Kurokawa T, Ogura J, Kobayashi M, Hirano T, et al (2011) In vitro and in vivo antioxidant properties of chlorogenic acid and caffeic acid. International Journal of Pharmaceutics 403: 136–138. 10.1016/j.ijpharm.2010.09.035 20933071

[8] Clifford MN (1999) Chlorogenic acids and other cinnamates—nature, occurrence and dietary burden. Journal of the Science of Food and Agriculture 79: 362–372.

[9] Gonthier MP, Verny MA, Besson C, Remesy C, Scalbert A (2003) Chlorogenic acid bioavailability largely depends on its metabolism by the gut microflora in rats. Journal of Nutrition 133: 1853–1859. 12771329 10.1093/jn/133.6.1853

[10] Tsai CM, Yen GC, Sun FM, Yang SF, Weng CJ (2013) Assessment of the Anti-invasion Potential and Mechanism of Select Cinnamic Acid Derivatives on Human Lung Adenocarcinoma Cells. Molecular Pharmaceutics 10: 1890–1900. 10.1021/mp3006648 23560439

[11] Gao RF, Fu YH, Wei ZK, Zhou ES, Li YM, Yao MJ, et al (2014) Chlorogenic acid attenuates lipopolysaccharide-induced mice mastitis by suppressing TLR4-mediated NF-kappa B signaling pathway. European Journal of Pharmacology 729: 54–58. 10.1016/j.ejphar.2014.01.015 24457123

[12] Xu YX, Chen JW, Yu XA, Tao WW, Jiang FR, Yin ZM, et al (2010) Protective effects of chlorogenic acid on acute hepatotoxicity induced by lipopolysaccharide in mice. Inflammation Research 59: 871–877. 10.1007/s00011-010-0199-z 20405164

[13] Frontela C, Ros G, Martinez C, Sanchez-Siles LM, Canali R, Virgili F (2011) Stability of Pycnogenol (R) as an ingredient in fruit juices subjected to in vitro gastrointestinal digestion. Journal of the Science of Food and Agriculture 91: 286–292. 10.1002/jsfa.4183 20872816

[14] Lopez-Nicolas R, Gonzalez-Bermudez CA, Ros-Berruezo G, Frontela-Saseta C (2014) Influence of in vitro gastrointestinal digestion of fruit juices enriched with pine bark extract on intestinal microflora. Food Chemistry 157: 14–19. 10.1016/j.foodchem.2014.01.126 24679746

[15] Paszti-Gere E, Szeker K, Csibrik-Nemeth E, Csizinszky R, Marosi A, Palocz O, et al (2012) Metabolites of Lactobacillus plantarum 2142 prevent oxidative stress-induced overexpression of proinflammatory cytokines in IPEC-J2 cell line. Inflammation 35: 1487–1499. 10.1007/s10753-012-9462-5 22476971

[16] Farkas O, Matis G, Paszti-Gere E, Palocz O, Kulcsar A, Petrilla J, et al (2014) Effects of Lactobacillus plantarum 2142 and sodium n-butyrate in lipopolysaccharide-triggered inflammation: comparison of a porcine intestinal epithelial cell line and primary hepatocyte monocultures with a porcine enterohepatic co-culture system. J Anim Sci 92: 3835–3845. 10.2527/jas.2013-7453 24987069

[17] Schierack P, Nordhoff M, Pollmann M, Weyrauch KD, Amasheh S, Lodemann U, et al (2006) Characterization of a porcine intestinal epithelial cell line for in vitro studies of microbial pathogenesis in swine. Histochem Cell Biol 125: 293–305. 10.1007/s00418-005-0067-z 16215741

[18] Repetto G, del Peso A, Zurita JL (2008) Neutral red uptake assay for the estimation of cell viability/cytotoxicity. Nat Protoc 3: 1125–1131. 10.1038/nprot.2008.75 18600217

[19] Farkas O, Palocz O, Paszti-Gere E, Galfi P (2015) Polymethoxyflavone Apigenin-Trimethylether Suppresses LPS-Induced Inflammatory Response in Nontransformed Porcine Intestinal Cell Line IPEC-J2. Oxid Med Cell Longev 2015: 673847 10.1155/2015/673847 26180592 PMC4477253

[20] Mohanty JG, Jaffe JS, Schulman ES, Raible DG (1997) A highly sensitive fluorescent micro-assay of H2O2 release from activated human leukocytes using a dihydroxyphenoxazine derivative. J Immunol Methods 202: 133–141. 9107302 10.1016/s0022-1759(96)00244-x

[21] Wang H, Joseph JA (1999) Quantifying cellular oxidative stress by dichlorofluorescein assay using microplate reader. Free Radic Biol Med 27: 612–616. 10490282 10.1016/s0891-5849(99)00107-0

[22] Paszti-Gere E, Csibrik-Nemeth E, Szeker K, Csizinszky R, Jakab C, Galfi P (2012) Acute oxidative stress affects IL-8 and TNF-alpha expression in IPEC-J2 porcine epithelial cells. Inflammation 35: 994–1004. 10.1007/s10753-011-9403-8 22083491

[23] Sakumoto R, Komatsu T, Kasuya E, Saito T, Okuda K (2006) Expression of mRNAs for interleukin-4, interleukin-6 and their receptors in porcine corpus luteum during the estrous cycle. Domest Anim Endocrinol 31: 246–257. 10.1016/j.domaniend.2005.11.001 16332426

[24] Hyland KA, Brown DR, Murtaugh MP (2006) Salmonella enterica serovar Choleraesuis infection of the porcine jejunal Peyer's patch rapidly induces IL-1beta and IL-8 expression. Vet Immunol Immunopathol 109: 1–11. 10.1016/j.vetimm.2005.06.016 16115691 PMC2613298

[25] Nygard AB, Jorgensen CB, Cirera S, Fredholm M (2007) Selection of reference genes for gene expression studies in pig tissues using SYBR green qPCR. BMC Mol Biol 8: 67 10.1186/1471-2199-8-67 17697375 PMC2000887

[26] Haller D, Bode C, Hammes WP, Pfeifer AMA, Schiffrin EJ, Blum S (2000) Non-pathogenic bacteria elicit a differential cytokine response by intestinal epithelial cell/leucocyte co-cultures. Gut 47: 79–87. 10.1136/gut.47.1.79 10861268 PMC1727962

[27] Reuter S, Gupta SC, Chaturvedi MM, Aggarwal BB (2010) Oxidative stress, inflammation, and cancer: How are they linked? Free Radical Biology and Medicine 49: 1603–1616. 10.1016/j.freeradbiomed.2010.09.006 20840865 PMC2990475

[28] Shin HS, Satsu H, Bae M-J, Zhao Z, Ogiwara H, Totsuka M, et al (2015) Anti-inflammatory effect of chlorogenic acid on the IL-8 production in Caco-2 cells and the dextran sulphate sodium-induced colitis symptoms in C57BL/6 mice. Food Chemistry 168: 167–175. 10.1016/j.foodchem.2014.06.100 25172696

[29] Langerholc T, Maragkoudakis PA, Wollgast J, Gradisnik L, Cencic A (2011) Novel and established intestinal cell line models—An indispensable tool in food science and nutrition. Trends in Food Science & Technology 22: S11–S20. 10.1016/j.tifs.2011.03.010 PMC7172287 32336880

[30] Francisco V, Costa G, Figueirinha A, Marques C, Pereira P, Miguel Neves B, et al (2013) Anti-inflammatory activity of Cymbopogon citratus leaves infusion via proteasome and nuclear factor-kappaB pathway inhibition: contribution of chlorogenic acid. J Ethnopharmacol 148: 126–134. 10.1016/j.jep.2013.03.077 23583902

[31] Melillo de Magalhães P, Dupont I, Hendrickx A, Joly A, Raas T, Dessy S, et al (2012) Anti-inflammatory effect and modulation of cytochrome P450 activities by Artemisia annua tea infusions in human intestinal Caco-2 cells. Food Chemistry 134: 864–871. 10.1016/j.foodchem.2012.02.195 23107701

[32] Hwang SJ, Kim YW, Park Y, Lee HJ, Kim KW (2014) Anti-inflammatory effects of chlorogenic acid in lipopolysaccharide-stimulated RAW 264.7 cells. Inflammation Research 63: 81–90. 10.1007/s00011-013-0674-4 24127072

[33] Shan J, Fu J, Zhao Z, Kong X, Huang H, Luo L, et al (2009) Chlorogenic acid inhibits lipopolysaccharide-induced cyclooxygenase-2 expression in RAW264.7 cells through suppressing NF-κB and JNK/AP-1 activation. International Immunopharmacology 9: 1042–1048. 10.1016/j.intimp.2009.04.011 19393773

[34] Du W-Y, Chang C, Zhang Y, Liu Y-Y, Sun K, Wang C-S, et al (2013) High-dose chlorogenic acid induces inflammation reactions and oxidative stress injury in rats without implication of mast cell degranulation. Journal of Ethnopharmacology 147: 74–83. 10.1016/j.jep.2013.01.042 23473868

[35] Soh N (2006) Recent advances in fluorescent probes for the detection of reactive oxygen species. Anal Bioanal Chem 386: 532–543. 10.1007/s00216-006-0366-9 16609844

[36] Garbetta A, Capotorto I, Cardinali A, D'Antuono I, Linsalata V, Pizzi F, et al (2014) Antioxidant activity induced by main polyphenols present in edible artichoke heads: influence of in vitro gastro-intestinal digestion. Journal of Functional Foods 10: 456–464.

