# Supplementary material for: Chlorogenic Acid Combined with Lactobacillus plantarum 2142 Reduced LPS-Induced Intestinal Inflammation and Oxidative Stress in IPEC-J2 Cells
Source: PLoS One. 2016 Nov 18;11(11):e0166642. doi: 10.1371/journal.pone.0166642 (PMC5115761; doi:10.1371/journal.pone.0166642)
Supplement: S1 Table — (DOCX) [file pone.0166642.s001.docx]

**S1 Table Viability of IPEC-J2 cells after chlorogenic acid treatment**

| **Absorbance after 1h treatment** |  |  |  |
| --- | --- | --- | --- |
| **Control** | **25 μM** | **50 μM** | **100 μM** |
| 0.11 | 0.113 | 0.104 | 0.063 |
| 0.102 | 0.1 | 0.106 | 0.063 |
| 0.089 | 0.096 | 0.097 | 0.059 |
| 0.088 | 0.112 | 0.103 | 0.054 |
| 0.087 | 0.106 | 0.1 | 0.094 |
| 0.092 | 0.093 | 0.103 | 0.068 |
| 0.106 | 0.105 |  | 0.058 |
| 0.105 | 0.104 |  | 0.061 |
| **Absorbance after 4h treatment** |  |  |  |
| 0.11 | 0.087 | 0.095 | 0.06 |
| 0.102 | 0.072 | 0.084 | 0.073 |
| 0.089 | 0.096 | 0.078 | 0.071 |
| 0.088 | 0.088 | 0.062 | 0.06 |
| 0.087 | 0.095 | 0.064 | 0.067 |
| 0.092 | 0.073 | 0.077 | 0.072 |
| 0.106 | 0.07 | 0.07 | 0.064 |
| 0.105 | 0.082 | 0.06 |  |
| **Absorbance after 24h treatment** |  |  |  |
| 0.11 | 0.088 | 0.071 | 0.066 |
| 0.102 | 0.089 | 0.071 | 0.066 |
| 0.089 | 0.093 | 0.066 | 0.067 |
| 0.088 | 0.0838 | 0.072 | 0.087 |
| 0.087 | 0.092 | 0.068 | 0.072 |
| 0.092 | 0.078 | 0.068 | 0.06 |
| 0.106 | 0.069 | 0.066 | 0.064 |
| 0.105 |  | 0.061 |  |
