# Supplementary material for: Chlorogenic Acid Combined with Lactobacillus plantarum 2142 Reduced LPS-Induced Intestinal Inflammation and Oxidative Stress in IPEC-J2 Cells
Source: PLoS One. 2016 Nov 18;11(11):e0166642. doi: 10.1371/journal.pone.0166642 (PMC5115761; doi:10.1371/journal.pone.0166642)
Supplement: S2 Table — (DOCX) [file pone.0166642.s002.docx]

**S2 Table Relative gene expression and protein concentrations of inflammatory markers**

| **Relative gene expression values** | | |  |  | |  | |  | |  | |  | |  |
| --- | --- | --- | --- | --- | --- | --- | --- | --- | --- | --- | --- | --- | --- | --- |
| **LPS 10 ug/ml** | | |  |  | |  | |  | |  | |  | |  |
| **IL6** | | | **IL8** | **TNFa** | | **COX-2** | | **CGA25 + LPS** | |  | |  | |  |
| 2.986 | | | 3.295 | 3.263 | | 4.492 | | **IL6** | | **IL8** | | **TNFa** | | **COX-2** |
| 1.925 | | | 2.424 | 5.539 | | 3.044 | | 1.025 | | 3.039 | | 2.016 | | 0.843 |
| 1.867 | | | 3.377 | 4.797 | | 6.34 | | 0.665 | | 0.964 | | 0.757 | | 0.615 |
|  | | | 4.37 |  | | 6.993 | | 0.577 | | 1.655 | | 2.342 | | 1.102 |
| **CGA 50+LPS** | | |  |  | |  | | **Lp 2142 + LPS** | |  | |  | |  |
| **IL6** | | | **IL8** | **TNFa** | | **COX-2** | | **IL6** | | **IL8** | | **TNFa** | | **COX-2** |
| 0.918 | | | 1.744 | 3.648 | | 1.413 | | 0.668 | | 1.071 | | 1.432 | | 0.527 |
| 0.623 | | | 1.467 | 1.302 | | 0.578 | | 0.895 | | 1.012 | | 2.536 | | 1.429 |
| 0.859 | | | 1.644 | 1.712 | | 0.947 | | 0.713 | | 1.041 | | 1.962 | | 1.662 |
|  | | |  |  | |  | |  | |  | | 1.906 | |  |
| **CGA 25+Lp2142+LPS** | | |  |  | |  | |  | |  | |  | |  |
| **IL6** | | | **IL8** | **TNFa** | | **COX-2** | |  | |  | |  | |  |
| 0.781 | | | 0.423 | 1.014 | | 0.381 | |  | |  | |  | |  |
| 0.406 | | | 0.524 | 2.162 | | 0.715 | |  | |  | |  | |  |
| 0.551 | | | 0.953 | 2.012 | | 0.892 | |  | |  | |  | |  |
| **Protein concentrations (pg/ml)** | | | | |  | |  | |  | |  | |  |  |
| **Control** | **LPS** | **LPS+** | | | **LPS+** | | **LPS+** | | **LPS+CGA 25 μM+** | | | |  |  |
|  |  | **CGA 25 μM** | | | **CGA 50 μM** | | **Lp2142** | | **Lp2142** | |  | |  |  |
| **IL-6, 6 h** |  |  | | |  | |  | |  | |  | |  |  |
| 781.5 | 1461.5 | 1131.5 | | | 686.5 | | 436.5 | | 329.8 | |  | |  |  |
| 673.2 | 1406.5 | 799.8 | | | 638.2 | | 369.8 | | 223.2 | |  | |  |  |
| 814.8 | 1139.8 | 709.8 | | | 663.2 | | 484.8 | | 164.8 | |  | |  |  |
| **IL-8, 24 h** |  |  | | |  | |  | |  | |  | |  |  |
| 80.8 | 96.2 | 74.6 | | | 77.7 | | 85.4 | | 86.9 | |  | |  |  |
| 79.2 | 93.1 | 75.4 | | | 84.6 | | 65.4 | | 85.4 | |  | |  |  |
| 75.4 | 94.6 | 76.2 | | | 77.4 | | 76.2 | | 82.3 | |  | |  |  |
