# Supplementary material for: Chlorogenic Acid Combined with Lactobacillus plantarum 2142 Reduced LPS-Induced Intestinal Inflammation and Oxidative Stress in IPEC-J2 Cells
Source: PLoS One. 2016 Nov 18;11(11):e0166642. doi: 10.1371/journal.pone.0166642 (PMC5115761; doi:10.1371/journal.pone.0166642)
Supplement: S3 Table — (DOCX) [file pone.0166642.s003.docx]

**S3 Table Fluorescence intensity values in the ROS measurements**

| **Fluorescence intesity values** | | |  |  |  |
| --- | --- | --- | --- | --- | --- |
| **Control** | **LPS** | **LPS+** | **LPS+** | **LPS+CGA 25 μM+** | |
|  |  | **CGA25 μM** | **Lp2142** | **Lp2142** |  |
| Amplex Red method(560nm/590nm) | |  |  |  |  |
| 48692 | 47054 | 21725 | 32348 | 24684 |  |
| 43705 | 43628 | 22663 | 31798 | 24676 |  |
| 44589 | 44066 | 23413 | 30023 | 22797 |  |
| 43685 | 46377 | 23399 | 29395 | 22852 |  |
| 42279 | 44454 | 22719 | 31150 | 16535 |  |
| 41568 | 43006 | 24066 | 29078 | 21799 |  |
| DCFH-DA method (480 nm/590 nm) | |  |  |  |  |
| 55761 | 90661 | 52607 | 41863 | 48200 |  |
| 57003 | 93998 | 56513 | 43012 | 50006 |  |
| 62808 | 108372 | 72204 | 57530 | 37010 |  |
| 64431 | 106354 | 75442 | 58826 | 42503 |  |
| 58886 |  | 74990 | 71270 | 74817 |  |
| 59682 |  | 75951 | 75688 | 84124 |  |
